# The Syndrome of Catatonia

**DOI:** 10.3390/bs5040576

**Published:** 2015-12-09

**Authors:** James Allen Wilcox, Pam Reid Duffy

**Affiliations:** 1 Department of Psychiatry, University of Arizona, 1401 E University, Tucson, AZ 85721, USA; 2 Tucson VA Medical Center, 3601 South 6th Avenue, Tucson, AZ 85723, USA; E-Mail: pamelareid.duffy@va.gov

**Keywords:** catatonia, psychosis, stupor

## Abstract

Catatonia is a psychomotor syndrome which has historically been associated with schizophrenia. Many clinicians have thought that the prevalence of this condition has been decreasing over the past few decades. This review reminds clinicians that catatonia is not exclusively associated with schizophrenia, and is still common in clinical practice. Many cases are related to affective disorders or are of an idiopathic nature. The illusion of reduced prevalence has been due to evolving diagnostic systems that failed to capture catatonic syndromes. This systemic error has remained unchallenged, and potentiated by the failure to perform adequate neurological evaluations and catatonia screening exams on psychiatric patients. We find that current data supports catatonic syndromes are still common, often severe and of modern clinical importance. Effective treatment is relatively easy and can greatly reduce organ failure associated with prolonged psychomotor symptoms. Prompt identification and treatment can produce a robust improvement in most cases. The ongoing prevalence of this syndrome requires that psychiatrists recognize catatonia and its presentations, the range of associated etiologies, and the import of timely treatment.

## 1. History and Commentary

Catatonia is a syndrome of motor dysregulation associated with a variety of illnesses. Bellack described derivation of the term from the Greek *kata* (down) and *tonas* (tension or tone) [1]. Papathomopoulus and Knoff offered another origin: that of *kata’s* alternate meaning (completely), which as a prefix strengthens the verb *tieno* (tension, stretching) and renders *katateino.* In early lectures, the syndrome was described in German as *Spannungsirresein,* to connote “the insanity of tension” [2]. Etymology aside, the hallmark of the syndrome catatonia is stupor accompanied by psychomotor disturbances. The Diagnostic and Statistical Manual (DSM­5) of the American Psychiatric Association documents a modern specification of the catatonic syndrome, and reports that catatonia can be found in a variety of disorders [3]. The DSM­5 criteria include the presence of three symptoms from the following list of twelve: stupor; catalepsy; waxy flexibility; mutism; negativism; posturing; mannerisms; stereotypy; agitation; grimacing; echolalia; and echopraxia. Other common symptoms are motor resistance to simple commands, posturing, rigidity, automatic obedience, and repetitive movements [3]. This specification is clinically useful, and is a significant improvement from that of DSM­lV.

Diagnostic parsimony has been long in coming. It is noteworthy that many signs and symptoms of catatonia have been reported. This may have been due, in part, to the growing science of psychiatry and zeal for naming and classifying numerous odd and bizarre behaviors. These early—and often colorful—descriptions of catatonia demonstrate that this symptom cluster has been recognized as a syndrome for quite some time. Prior to the late nineteenth century, several terms were used to describe conditions characterized by stupor alternating with excitement in the English medical literature. Early case reports shared a common theme of psychosis with psychomotor symptoms.

**Figure 1:**
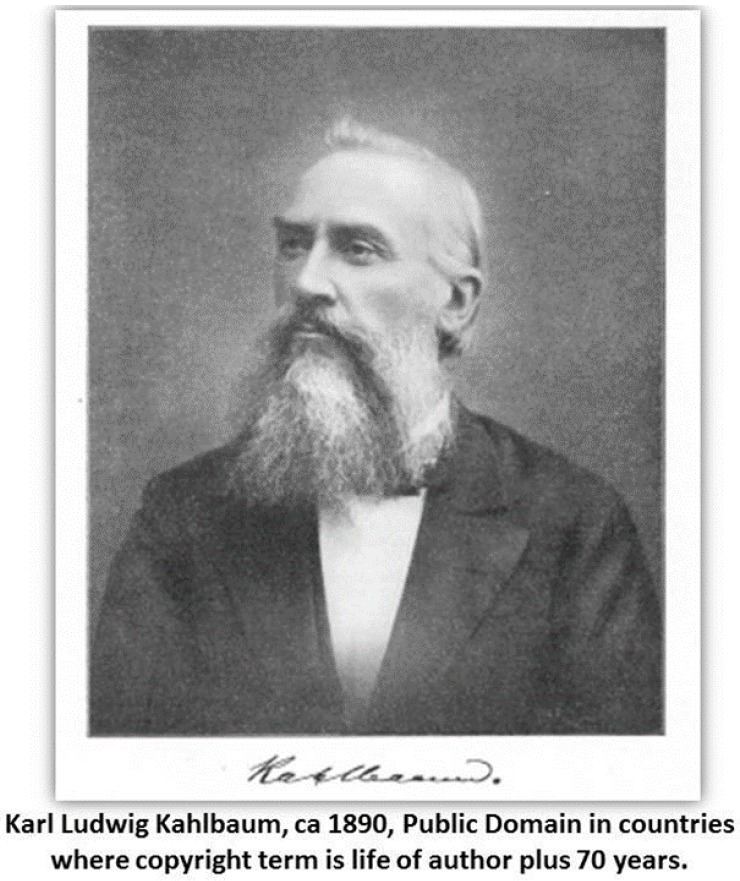
Karl Ludwig Kahlbaum.

Many years would pass before the science of descriptive psychopathology evolved for a clearer picture of catatonia. Most clinical historians would agree that Karl Kahlbaum (see Figure 1) conducted the first disciplined and systematic inquiry that would eventually define catatonia as a discrete syndrome**.** He followed a group of patients from his practice at the Riemer Sanitarium in Germany during the late 19th century. His early descriptions of the condition concentrated on motor symptoms of mutism, catalepsy (waxy flexibility), verbigeration, stereotypies, and negativism [4]. The key symptoms of catatonia are given in Table 1. It is clear that in addition to Kaltbaum’s important specification of the catatonic syndrome, his work supported multicausality and did not consider its presentation as indicative of a single disease entity. He described it in a variety of patients with different primary conditions including depression, mania, and overt psychosis. He presented this work in theoretical lectures as early as 1866, and in a classic monograph in 1874 [4]. Many clinicians modeled their concept of the syndrome after his description, and identified the syndrome co-occurring with a diverse variety of disorders.

Though early clinicians began to recognize catatonia, its clinical picture was confounded by observations of patients with chronic, deteriorating illnesses. This shared understanding reflected available evidence of the times: that catatonia led to a uniformly poor outcome, similar to a dementing disease. When Kraepelin considered the condition, recognition of variability among possible courses was precluded. This was not unreasonable, given the state of the science, severity of illness(es) resulting in institutionalization, and paucity of effective treatments for mental disorders. The sampling of available cases to those of hospitalized patients created a bias towards inevitable decline. Kraepelin noted the deteriorating course of his patients and concluded that catatonia was one of three primary forms of dementia praecox, along with paranoid and disorganized types [5].

**Table 1:** Features of catatonia.

Symptom	Description of pathology
Stupor	decreased response to external stimuli, hypoactive behavior
Immobility	akinetic behavior, resistance to being moved
Waxy flexibility	slight resistance to being moved
Mutism	verbally unresponsive, refusal to speak
Posturing	purposely maintaining a position for long periods of time
Excitement	frantic, stereotyped or purposeless activity
Echolalia	senseless repetition of the words of others (echolalia)
Echopraxia	mimicking the movements of others
Staring	eyes fixed and open for long periods of time
Catalepsy	the passive adoption of a posture

Eugene Bleuler reorganized the criteria for dementia praecox and included milder, less chronic presentations [6]. Having distinguished it from uniformly progressive disorders, he renamed the condition schizophrenia. Akin to Kraepelin’s classifications of dementia praecox, Bleuler’s concentration on disturbances of ambivalence, autism, and affect resulted in the inclusion of the catatonic syndrome as a subgroup of schizophrenia. While Kraepelin continued to define catatonia as a type of dementing illness, both he and Bleuler recognized that the syndrome could be found in different types of psychosis [5,6]. Other clinicians increasingly identified that catatonia was a medical condition that occurred in many settings [5,6,7,8].

The next major development towards a modern understanding of catatonia proceeded as more clinicians recognized its association with affective disorders. By 1913 Kirby had reported clear cases of catatonia in patients with manic depressive illness [8], and early work by Kleist supported the notion of catatonia’s association with mood disorders [9]. An emerging body of work supported recognition that catatonia was not merely a form of schizophrenia, but could be found in mood disorders as well as in a variety of medical conditions [8,9,10,11,12]. Again, this progress towards informing our modern knowledge of catatonia—specifically, its common association with affective illness—was left relatively unnoticed, likely overshadowed by interest in infectious diseases affecting the brain.

These conditions could confound the inexperienced clinician by the nature and severity of their psychomotor symptoms. Early prospective studies revealed that untreated catatonia had a high mortality rate in a variety of settings [9]. This would easily be accounted for by underlying medical conditions.

In spite of an evolving consensus that catatonic syndromes were oft associated with medical illnesses, clinicians from the psychoanalytic school remained focused on analytic explanations. Rigorous medical examinations were often neglected. A review of the analytic literature reveals case reports of catatonia, but few scientific studies. Consideration of catatonia as a defense mechanism with sensorimotor regression was a likely psychoanalytic interpretation. Analytical concepts of catatonia were virtually impossible to confirm [13,14]. Not surprisingly, early psychoanalysts had difficulty treating catatonia.

Kahlbaum’s descriptive work developed to include longitudinal observations of his patients. This work was compromised, however, by common institutional diseases of the era, such as pneumonia and tuberculosis—which made long term follow-up of the natural history of the catatonic syndrome very difficult. Similarly, the works of Kraepelin and Hoch documented attempts to collect longitudinal data, but these efforts suffered from limitations of the medical therapies of the time as well [5,11,15,16].

Gjessing described a condition he referred to as periodic catatonia in the 1930s [17]. Gjessing quoted Kraepelin as estimating that about 2% of admissions to mental hospitals in the early 20th century would be of this type [7,17]. This rare variety of the syndrome was characterized by phases of catatonic symptoms that quickly alternated with completely asymptomatic periods [17,18]. Gjessing reported cases where patients were normal one day, then catatonic the next—only to return to an asymptomatic phase the following day. Case reports of recurrent brief episodes of catatonia followed by total remission [19,20] remain in the literature today.

Treatment of periodic catatonia was often unusual. Gjessing was among clinicians testing treatments for catatonia. He followed patients over a period of years, and considered them to be candidates for metabolic studies [17]. His research detected changes in blood nitrogen levels that correlated with stages of stupor and excitement [17,18]. Gjessing found a benefit in treating many of this patients with thyroxine in addition to electroconvulsive therapy [18]. In modern times, the use of thyroxine in catatonia has nearly disappeared. The notions of cyclic changes in nitrogen balance have largely been abandoned. Some research has suggested that periodic catatonia may have a unique genetic basis [19,20]. This is supported by results from family studies [19]. Genetic analysis indicates possible loci at chromosome 15q15 and chromosome 22q13 [20].

Early treatment involved little more than supportive care. Feeding was done by nasogastric tubes and body temperature was regulated with cold baths. Treatment for the syndrome evolved with the use of barbiturates and electroconvulsive therapy (ECT) in the late 1920s [10]. As use of tranquilizers and ECT became increasingly popular treatments for all psychiatric disorders, it became clear that they were uniquely beneficial to catatonic patients. At first, intravenous barbiturates were used to conduct interviews as the patients became more lucid for 20–30 min after the injection [10,16]. These windows of lucidity also allowed time to address severely ill patients’ dietary and hygienic needs.

## 2. Prognosis/Course of Catatonia

As more effective medical treatments were established both for the catatonic syndrome and the concurrent diseases historically found with it, the ability to do cohort studies on a level field with other medical conditions eventually became possible. Hamilton, in 1962 [10], observed that catatonia seemed to have two kinds of outcomes, dependent on its affective or psychotic comorbidity. He reported that when associated with mood disorders, it followed an episodic course and often went into full recovery for long periods of time. On the other hand, he noted that catatonia associated with schizophrenia typically went into protracted periods of stupor, interrupted by alertness that was nonetheless characterized by poor hygiene, asocial behavior, and disorganized activity. Of the catatonic patients diagnosed with schizophrenia, Hamilton reported about 32% improved significantly over time, while the other 68% continued on a deteriorating course with early demise [10]. Publications of other studies of prognosis reported recovery in only 20%­40% of cases, with the remaining cases going on to continuing psychosis and disintegration of the personality. As in previous reports, patients with affective disorders fared better than those diagnosed with schizophrenia. However, work published as late as 1966 demonstrated a high overall mortality for catatonia, even when modern treatments were available [12].

In the early 1970s a large retrospective cohort was investigated by physicians at the University of Iowa. This was made possible by the Iowa Psychopathic Hospital, where records were preserved for almost 20,000 psychiatric patients hospitalized between 1920 and 1934. The patients’ signs and symptoms were carefully documented in these records, yielding rich and detailed descriptions of behavior. Such exceptionally specific information allowed for reconstruction and retrospective diagnosis using the Research Diagnostic Criteria (RDC) [21]. The assessments were found to have very high kappa coefficients or inter­rater reliability [22,23]. In addition to analysis of data from chart reviews, the Iowa patients themselves could frequently be contacted, and followed over time to monitor the course and progression of their illness. As the hospital’s catchment area was a primarily rural environment, many families were linked to their geographic location for their livelihood—often farming. This made the patients less likely to relocate, available to contact over decades. Patients born at the end of the 19th century could be followed through much of the 20th century with remarkable ease. In some cases, surviving family members were available to provide additional histories of the patients’ functioning, adding to the wealth of data available to categorize the course of illness and recovery.

From the resultant analyses of this rich data base, the Iowa group provided compelling evidence that cases of catatonia were associated with affective illness with similar frequency as with schizophrenia. Morrison selected charts from the Iowa project for further study. He focused on catatonia and found very interesting outcomes: In his group of catatonic subjects, 40% were found to have recovered [23]. The factors associated with improvement were diagnosis of mood disorder, acute onset, and treatment with electroconvulsive therapy [19,20].

Catatonia remained an epidemiological enigma. After Kahlbaum’s initial description of the syndrome, the prevalence of catatonia seemed stable for several decades. In the years 1900 to 1920 catatonia was consistently found in 10% to 40% of admissions to psychiatric hospitals [23]. In the decades to come, however, it was believed that catatonia was declining in occurrence and on the verge of becoming a rare syndrome.

By the 1970s, clinicians began trying to determine the cause of this perceived change [24,25,26,27,28,29]. Several hypotheses were put forth to explain this phenomenon. The perceived decrease in hospital admissions of patients with catatonic features could be related to improvements in the detection and treatment of the primary illnesses potentially leading to the catatonic state. In addition, it was noted that modern treatments of underlying etiologies may have preempted the occurrence of catatonia in otherwise susceptible patients.

A similar hypothesis considers many cases of catatonia to be related to ineffective treatment of affective disorders [28]. Thus, the decline in hospital admissions for catatonia may be attributed to the increase in efficacy of outpatient treatments for disorders of mood [28]. The discovery of effective treatments for affective illnesses has significantly evolved over the past 60 years, the same time frame in which a decline of hospital admissions for catatonia was observed. It should be noted that electroconvulsive therapy has been effective in the treatment of catatonia in affective disorders for quite some time, as early as 1929 [10]. Increasingly, clinicians found patients with catatonia followed prospectively did indeed have affective disorders [8,11,15].

The works of Taylor, Abrams, and Fink demonstrated a strong tendency for catatonia to occur in psychotic depression, as well as in mania [15]. This finding seemed to rebuff the idea of catatonia arising from the delayed treatment of more common psychiatric illness, since treatments for affective disorders have existed for quite some time. A young age of onset for psychiatric disease was noted as a risk factor for the catatonic syndrome by Ungvari *et al.* [29]. This would also seem to counter the argument that delayed treatment caused catatonia. In another cohort study of 568 cases of catatonic patients, Kleinhaus and Harlap found an association with the schizophrenic diagnosis [30]. This may have been due to a selection bias in a sample with many psychotic patients.

Common theories to explain decreased prevalence of catatonia have included: use of increasingly-restrictive definitions of the syndrome; more reliable exclusion criteria; and improved treatment resulting in fewer cases reaching the catatonic stage associated with advanced illness [29,30,31,32,33]. Employment of alternative classification systems such as the Leonhard approach have yielded significantly more cases of catatonia than the use of RDC [21]. It is also possible that some “false negatives” occurred in which true cases of catatonia were mistaken for neuroleptic malignant syndrome. Clinicians noted a tendency for catatonic episodes to last at least six months in almost all cases studied, as well as multiple hospital admissions [30,33]. All of these variables may contribute to the underestimation of catatonia in clinical systems.

## 3. Malignant Catatonia

A subgroup of catatonic patients develop severe level of metabolic decompensation [34]. For reasons that are not entirely clear, this condition has developed its own niche in psychiatric terminology. This is not merely a matter of nosology, since these cases are quite severe and can have lethal outcomes. Many clinicians fear that malignant catatonia is the final common pathway of poorly understood, intractable cases [33,34]. Patients unresponsive to treatment progress to lethal metabolic conditions. However, the astute clinician can minimize harm. Rapid support and therapeutic intervention (usually with ECT) can be lifesaving.

Patients in the chronic excited phase are clinically challenging. They easily become exhausted and are at risk of injury to themselves and/or caregivers. These patients can develop rhabdomyolysis, fever, and kidney failure. These patients are at risk for heart failure as well [34]. Treatment for such patients usually involves electroconvulsive therapy, supportive metabolic care and, possibly, judicious sedation with a barbiturate or lorazepam [33,35].

The immobile, rigid catatonics are often at risk of the malignant syndrome. While standing, sitting, or lying down for extended periods of time, they develop pooling of lymphatic and vascular fluids, with possible separation of proteins from aqueous serum. Immobile catatonics are at risk for pulmonary emboli, edema, and dehydration of moist membranes. Dry sclera puts the eyes at risk for ulceration and infection. Protracted rigidity leads to rhabdomyolysis and kidney failure. Many cases of immobile catatonia develop fever. While malignant catatonia may be as much an adjective as it is a diagnostic term, it is always serious and potentially life threatening.

Some cases of malignant catatonia develop from inadequate treatment of the early phases of catatonia, whereas others develop quickly [36,37]. Most clinicians currently advise immediate intervention with electroconvulsive therapy [10]. The cases as described above rarely respond to other interventions and continue to deteriorate on supportive care alone [34].

## 4. Differential Diagnosis

The differential diagnosis of catatonia is extensive. Many conditions present with movement dysregulation and stupor. It is vital to perform a comprehensive medical examination and detailed neurological evaluation upon first contact with the patient. This should be accompanied by blood work, including a complete metabolic panel, blood count and analysis, electrolyte, kidney and liver function tests, a toxicology screen, as well as a battery of common hormonal values. A brain MRI and an electroencephalogram (to rule out non-convulsive status epilepticus) are required. Some cases with fever or elevated neutrophil count will require examination of cerebrospinal fluid.

After these examinations, a useful screening test like that developed by Peralta could be used to evaluate psychiatric symptoms [38]. With these methods, most cases of catatonia can be safely assessed and treated. Gelenberg published a most detailed differential diagnosis pathway in 1976 [39]. 

The organic conditions which present with catatonia are many, and may be innumerable***.*** Such a list may include, but is not limited to those listed here***.*** Metabolic conditions include: diabetic ketoacidosis [40]; parathyroid adenoma [41]; pellagra [42]; and homocystinuria [43]. Neurological disorders can result in catatonia; akinetic mutism [44] is an excellent example.

Fink recommended that in the presence of normal laboratory values, a lorazepam change test can provide a confirmatory diagnosis [45]. Most cases of catatonia will respond to 2–4 mg of intravenous lorazepam. The injection will usually bring about normal motor activity and restore mental clarity within one to two min. This reduction in symptoms is brief, lasting 20–30 min before the patient gradually returns to a rigid stupor [45]. It is very important that a breathing apparatus is available, and diligent clinical vigilance is used to prevent respiratory depression or asphyxiation.

## 5. Neuroleptic Malignant Syndrome

Among the differential entities, a discussion of Neuroleptic Malignant Syndrome (NMS) is warranted. This severe medical condition is brought about by the use of antipsychotic medication [46], and was first reported in 1956 [47]. It has been associated with virtually all conventional antipsychotics and some atypical antipsychotics. The historical incidence of NMS has been about 2% [46]. 

Patients with NMS typically present with fever, muscle rigidity and altered mental status [46,48]. In some cases, akathisia, is sometimes seen rather that rigidity. Patients may develop delirium and mutism during the course of NMS and can appear much like cases of idiopathic catatonia. White blood cell count and CPK often increase in NMS and rhabdomyolysis is frequently seen. Hypertensive crisis and metabolic acidosis are common. The fever is thought to be caused by dopamine blockade of the hypothalamus [48]. It is thought that calcium from the sarcoplasmic reticulum may be the cause for muscle rigidity [48]. 

Neuroleptic Malignant Syndrome is a medical emergency. It can lead to death if not treated. Most patients will need the support of an intensive care unit to provide for adequate hydration, ventilation, and temperature regulation [46]. Pharmacological treatments have not been uniformly successful. Bromocriptine and dantrolene have been found to be of benefit in some cases [46]. Early studies have reported mortality rates as high as 30%, but recent data suggests that early recognition and treatment can reduce this to less than 10% [48].

Some authors have suggested that catatonia and NMS may be disorders of the same basic spectrum, but the data for this position is less than vigorous. The similarity of presentation, high potential for mortality, and occurrence in similar populations (psychotic patients) makes the importance of clinical vigilance all the more vital. 

## 6. Treatment

The first order of care for the individual with catatonia is to establish the medical etiology. As described above, in the case of idiopathic catatonia, a simple reduction in catatonic symptoms is to be desired. Immediate discontinuation of neuroleptics is indicated. While a very small number of cases of success with clozapine have been reported [49,50], it best to avoid use of antipsychotic medications altogether. Most of the neuroleptic drugs routinely prescribed for psychosis are likely to worsen the psychomotor symptoms of catatonia [10,16]. A small body of literature suggests that transcranial magnetic therapy may be useful in some patients [51].

Regardless of etiology, the care of the patient includes adequate hydration and other supportive measures. Concurrent care may include nutrition, cooling, prevention of aspiration, and consideration of thrombophlebitis prophylaxis. Once these measures have been taken, treatment of any underlying conditions should alleviate the psychomotor symptoms. This approach, however, does not manage all cases as catatonia may be idiopathic. Once medically stable, the active treatment usually falls into two categories, benzodiazepine activation or electroconvulsive therapy [52]. Most cases will respond quite well to large doses of lorazepam (2–5 mg), given intravenously, with care to monitor ventilation. If benzodiazepine therapy fails, or if the patient is in an emergent situation of lethal catatonia, electroconvulsive therapy is the likely treatment of choice [52]. A course of nine to twelve bilateral treatments is usually sufficient to reduce catatonic symptoms. After the ECT is completed oral lorazepam is introduced. In order to have a persistent response, most patients require several sessions of electroconvulsive treatment. Once ECT is complete, maintenance with lorazepam and a mood stabilizer such as valproic acid is often the most successful form of outpatient care.

In order to get a proper perspective of the presentation and treatment of catatonia it is useful to examine actual cases. 

### 6.1. Case #1

The patient was a 37 year old single white male with a history of chronic bipolar disorder. He had no prior history of catatonia, but did have a history of psychotic mania in the past two years. His family brought him to the hospital after an episode of depression progressed to refusal of meals, mutism, and refusal to leave his bed. Initial examination found a resistive, silent male with poor eye contact. He could only leave his chair with assistance and became rigid when physical assistance was offered. The patient offered passive resistance to attempts to move his arms and kept the arms posed in position for 5 to 10 min after they were moved (waxy flexibility).

His skin was sebaceous and ruddy in color. Pupils were reactive to light and accommodation. Reflexes were decreased, but equal bilaterally. Babinski’s sign was normal. Attempts to move limbs were met with resistance (gegenhalten). An electrocardiogram has normal. Urine toxicology was normal. Routine CBC, electrolytes, BUN, creatinine, and liver function tests were unremarkable. The patient had normal temperature and respirations. Blood pressure was 132/79. Levels of creatinine phosphokinase (CPK) were normal, suggesting that rhabdomyolysis had not started. Brain Magnetic Resonance Imaging revealed no abnormality. Chest X-ray was clear. Patient was not taking any psychiatric medications. Analysis of cerebrospinal fluid found no abnormalities. This patient met the criteria for catatonia in DSM­V. He was given a test dose of 3 mg intravenous lorazepam. Within sixty seconds of the injection, the patient sat up in bed and asked for something to eat. He held a logical conversation for ten min, and then gradually became disorganized and eventually mute. Within a few more min he became motionless and eventually rigid. Attending physicians felt that ECT was the treatment of choice. A series of spinal X-rays, electrocardiogram, and legal permission for ECT were obtained. Once electroconvulsive treatment began, a remarkable response was noted. The patient received his first treatment of bilateral ECT with setting of 150 volts at 0.5 s in a routine procedure.

After his first treatment, the patient was responsive to conversation for about three hours. He continued to receive this treatment every other day until nine sessions were completed. Cognition and rigidity improved with each treatment. After the last treatment, the patient was alert, conversant, and able to ambulate. His family related that the patient was “back to his old self”.

He was discharged on lorazepam 2 mg, three times a day along with fluoxetine 20 mg per day and Valproic acid 1000 mg at bedtime. Subsequent follow up a year later found him in recovery, and functioning well in his community.

### 6.2. Case #2

The second patient was a 52 year old single white female, who had been diagnosed with schizoaffective disorder for 30 years. This patient had had three prior episodes of catatonia; one at age 28, a second at age 34, and again at age 42. She presented with a history of one week of excited, purposeless activity. During the past week, she had repeated unintelligible words and phrases for hours and moved rapidly around a small area (six square feet) in her home. Throughout her evaluation in the ER she repeatedly yelled “peanut butter, ice cream, peanut butter, ice cream” for several hours. CBC revealed an elevated neutrophil count and a total WBC of 16,500. CPK was elevated. Chest X-ray and brain MRI were unremarkable. Liver enzymes and electrolytes were within normal limits. Her neurological exam was normal. Results of analysis of cerebrospinal fluid were normal. She was sent to the psychiatric unit and given 3 mg IV lorazepam, and became somewhat calmer for a few min, but changed very little. She was given 5 mg of olanzapine by mouth with no effect. The patient had to be spoon fed in a laborious process, with much of the food dropped and smeared on furniture. After four days on the unit the patient slowed down and eventually assumed a stiff catatonic posture. Her purposeless verbalizations stopped and she became mute.

Repeat chest X-ray was normal as was the repeat of her EKG and brain MRI. Spinal X-rays were normal. Legal consent was obtained to start electroconvulsive treatment (ECT). After one treatment of 150 volts for 0.5 s, the patient returned to a “normal” appearance. She was calm and spoke with a normal rate and content. She received five more ECTs and was discharged home on lorazepam.

## 7. Conclusions

Catatonia is a syndrome caused by a variety of brain diseases. It is likely to have been a part of the human pathology for centuries. Systematic observations began to appear in the literature beginning with Kahlbaum. His detailed descriptions allowed other clinicians to study the syndrome, culminating in the development of our current understanding. Clinicians and scientists have learned a considerable amount by study of this syndrome’s epidemiology. Early work was complicated by medical comorbidities and institutional conditions. Many early cases were lost to follow up. By the 1950s, the life course of patients with catatonia could be followed from onset to death. Treatments for catatonia, such as electroconvulsive therapy and barbiturates, were introduced early in 20th century [16,17]. Ongoing research revealed many causes for this syndrome. Catatonic patients fall into several general groups. Of these, those with affective symptoms may experience long periods of remission. Cases with the diagnosis of schizophrenia tend to have a more chronic course, but some of this may be due to treatable Neuroleptic Malignant Syndrome. Based on this review, we encourage psychiatrists to recognize catatonia as a syndrome, rather than a rare disease entity. Our old-school photos aside, awareness of catatonia is of modern clinical importance. Numerous studies have described the prevalence among psychiatric patients ranging from 7.6 to 38% [53,54]. Clinicians need be familiar with catatonia, its underlying medical conditions, as well as its idiopathic presentation. A proper clinical history and prompt treatment are a matter of life and death.
